# Reports of Injury Risks and Reasons for Choice of Sleep Environments for Infants and Toddlers

**DOI:** 10.1007/s10995-019-02803-7

**Published:** 2019-06-27

**Authors:** N. J. Scheers, Chauncey Dayton, Mary Batcher, Bradley T. Thach

**Affiliations:** 1 BDS Data Analytics, 5823 Jane Way, Alexandria, VA 22310 USA; 2 BDS Data Analytics and University of Maryland, College Park, USA; 3 Washington University School of Medicine, St. Louis, MO USA

**Keywords:** Crib injuries, Accidental suffocation, Safe sleep environment

## Abstract

**Objective:**

Compare mothers’ reports of injuries for infants and toddlers sleeping with crib-bumpers/mesh-liners/no-barriers and reasons for these sleep environment choices.

**Methods:**

A cross-sectional survey of mothers subscribing to a parenting magazine and using crib bumpers (n = 224), mesh liners (n = 262), and no barriers (n = 842). Analyses of four possible injuries (face-covered, climb-out/fall, slat-entrapment, hit-head) including multivariate logistic regression adjusted for missing data/demographics and Chi squared analyses of reasons for mothers’ choices.

**Results:**

Maternal reports of finding infants/toddlers with face covered had 3.5 times higher adjusted odds (aOR) for crib bumper versus mesh liner use. Breathing difficulties and wedgings were reported for infants/toddlers using crib bumpers but not mesh liners. Climb-outs/falls showed no significant difference in aORs for crib bumpers versus no-barriers and mesh liners versus no barriers. Reports of slat-entrapment were less likely for mothers using crib bumpers and mesh liners than using no barrier (aOR = .28 and .32). Reports of hit-heads were less likely for crib bumpers vs no barrier (aOR = .38) with no significant difference between mesh liners versus no barrier use. Mothers using crib bumpers and mesh liners felt their choice prevented slat-entrapment (89%, 91%); 93.5% of crib bumper users felt their choice prevented hit-heads. Significantly more mesh liner than crib bumper users chose them because “There is no suffocation risk” (64.1% vs. 40.6%), while 83.6% of no-barrier users chose them because “I was concerned about suffocation risk.”

**Conclusions for Practice:**

Mothers appeared to be more concerned about preventing minor risks than suffocation. Understanding reasons for mothers’ use of barriers/no-barriers is important in tailoring counseling for mothers with infants/toddlers.

**Electronic supplementary material:**

The online version of this article (10.1007/s10995-019-02803-7) contains supplementary material, which is available to authorized users.

## Significance Statement

*What is already known on this subject?* Although discouraged by the American Academy of Pediatrics as hazardous, soft bedding use in infants’ and toddlers’ cribs continues to be common. A recent study found that bumpers were the most frequent soft bedding use reported in 13 states and NYC.

*What this study adds?* This is the first study to compare the relative safety of crib bumpers, mesh liners, and no barrier in the crib by examining four injury risks for a general population of mothers. This is also the first study to examine mothers’ reasons for their choice of barriers/no barrier and the prevalence of those choices.

## Introduction

Sudden unexpected infant death accounts for ~ 3700 U.S. deaths annually from sudden infant death syndrome (SIDS), accidental suffocation and strangulation in bed, and unknown cause (Lambert et al. 2018). Soft bedding is a risk factor for SIDS (Hauck et al. 2003; Scheers et al. 1998; Kemp et al. 1994) and suffocation (Kemp et al. 1998; Colvin et al. 2014). The American Academy of Pediatrics (AAP) recommended against soft bedding use in infants’ sleeping environments as early as 1996 (AAP Task Force 1996). While a recent study found a decline in soft bedding use, from 85.9 to 54.7% (Shapiro-Mendoza et al. 2015), use remains fairly common (Moon and Hauck 2015). A recent survey of mothers from 13 states and New York City (NYC) found that 38.5% reported the use of any soft bedding (Bombard et al. 2018). Crib bumpers were the most frequently reported at 19.1%, ranging from 28% in NYC/New Jersey to 12% in Maryland that banned the sale of bumpers in 2013 (Md Code Reg. 2013). In advertisements marketed to parents and on web sites (Joyner et al. 2009; Kreth et al. 2017), bumpers were shown in 85% of magazine pictures with cribs.

Although implicated in suffocation deaths and injuries (Scheers et al. 2016; Thach et al. 2007) and discouraged by the AAP (Moon et al. 2016), crib bumpers remain popular with parents. While there is substantial data on the hazards inherent in the use of bumpers, to our knowledge there are no data on potential hazards of alternative barriers such as mesh liners or the use of no barriers in cribs.

**Fig. 1:**
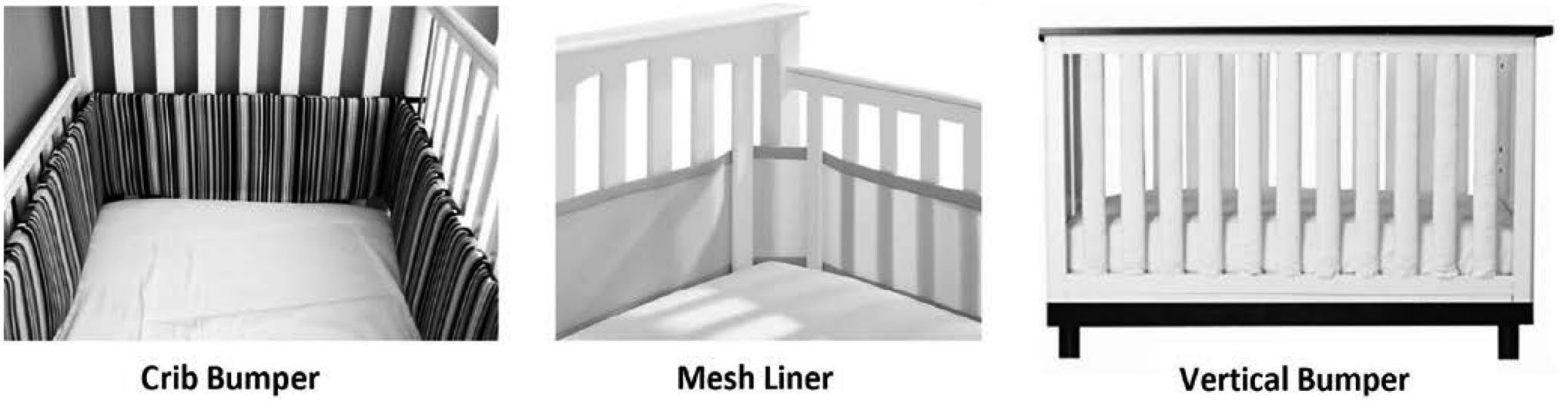
Three types of crib barrier

Our objective was to compare injury risks of crib bumpers with alternative products (Fig. 1) and no barriers in cribs through a survey of mothers who subscribed to a national parenting magazine. We chose to solicit subscribers to a national magazine that focused on issues of interest to mothers with infants and toddlers, speculating that this population would be more informed about AAP’s safe sleep recommendations.

We focused on four crib injuries: face-covered by a crib barrier; climb-outs and falls from cribs; slat-entrapment; and hitting-heads against the crib sides (Scheers et al. 2016). A secondary objective was to compare reasons for mothers’ choice of crib bumpers/mesh liners/vertical bumpers/no barrier (Ajao et al. 2011; Moon 2007; Pease et al. 2017) and the prevalence of their choices. Finally, we were interested in whether a population of mothers who subscribed to a parenting magazine, and thus more likely to be informed about AAP’s safe sleep recommendations, was actually compliant with them.

## Methods

### Participants

Using the internet-based SurveyMonkey survey program (https://www.surveymonkey.com/), we contacted 43,865 subscribers to Pregnancy & Newborn magazine (P&N) (https://www.pnmag.com/) using their subscriber email list after removing non-U.S. and organization addresses. We were unable to screen this list by any inclusion criteria because P&N had no information about individual subscribers. Thus, the list would be expected to include an unknown proportion of ineligible respondents such as corporations, fathers, grandparents, and mothers with no current infant/toddler.

Subscribers were offered an incentive to respond to our web survey’s email link by entering a lottery for one of ten $100 Amazon gift cards. Five pilot tests with different email subject lines and instructions for 100 randomly selected P&N subscribers found no significant differences in response rates. After three follow-up emails, 12,332 emails were opened and 4070 (33%) were responded to (AAPOR 2016). Inclusion criteria resulted in 1344 eligible respondents who were mothers of singleton infants/toddlers, ≤ 24 months old (Yeh et al. 2011), sleeping in cribs (Scheers et al. 2003) using a crib bumper (CB), mesh liner (ML), vertical bumper, or nothing (no barrier, NB). Mothers reporting vertical bumpers use (n = 16) were excluded due to the small sample size. Our response rate could have been substantially reduced because anyone opening our email solicitation could have noted the focus on mothers with infants/toddlers and not continue to submit the survey. About 66% of those starting the survey were ineligible since they were not a mother or did not have a qualifying infant/toddler. An accredited Institutional Review Board certified this study as an exempt survey (45 CFR 46.101(b)).

### Measures

A structured online questionnaire used multiple branching, skip patterns and comment fields with mean completion time = 16.03 min (median = 6.08). Questions included mothers’ reports of injuries (Online Appendix 1) and reasons for use described below. Pilot testing used one-on-one cognitive interviews lasting up to 2 h with five mothers who were paid $20: one using a bumper, one a mesh liner, three with nothing in the crib.

Mothers rated reasons for their choice of barriers/no barriers using a 5-point scale from “Strongly Agree” to “Strongly Disagree. “Strongly Agree” and “Agree” were combined to form a high rating. Mothers using crib bumpers, mesh liners, and vertical bumpers rated the same questions; mothers using no barrier received parallel questions.

### Analysis

Statistical analyses used SPSS (IBM Corp. 2017) with significance levels set at .05 and 95% confidence intervals (CIs). Comparisons among use-groups (CB/ML/NB) used Chi squared (χ^2^) tests and unadjusted odds ratios (uOR). Multivariate logistic regression (LR) models calculated the odds of an injury incident occurring adjusting for six demographic variables (aOR). For face-covered incidents, CB was compared to ML; otherwise, we used NB as the reference group (Dayton and Scheers 2018).

Adjustor variables in LR models were mother’s age, education, parity, race/ethnicity as well as infant/toddler’s age and sex. We used infant/toddler’s age reported for each injury incident when present and age at time of survey when not present, similar to time-to-event in survival analysis (Hosmer et al. 2008). Because ~ 16% of the cases had missing responses for one or more adjustor variables, consistent with recent pediatric studies, (Colvin et al. 2014; Lagon et al. 2018) we used the Markov Chain Monte-Carlo (MCMC) method in SPSS to create a pooled data set from 15 imputed data sets (Stuart et al. 2009).

## Results

### Study Population Characteristics

The study population consisted of 1328 mothers, 16.8% of whom used CBs (n = 224), 19.7% used MLs (n = 262), and 63.3% used NB (n = 842). About 35% were < 30 years old, 52% had ≥ college degree, 69% were White non-Hispanic, and 47% reported having one child. About 53% of the infants/toddlers were male and 47% were ≤ 12 months old. Compared to mothers using MLs and NB, mothers using CBs were more likely to be younger, less educated, be Hispanic or other minority, and have more children. There was no significant difference among use-groups for infant/toddler’s sex or age at time of the survey (Table 1).

**Table 1:** Study population characteristics by use-group

Characteristics	Use-group			Total	p^+^
	Crib bumpers	Mesh liners	No barrier		
Maternal age (years), n =	177	224	746	1147	0.049
≤ 24	15.8%	9.4%	8.8%	10.0%	
25–29	20.9%	22.3%	26.0%	24.5%	
30–34	32.8%	42.9%	39.9%	39.4%	
35+	30.5%	25.4%	25.2%	26.2%	
Maternal education, n =	181	223	751	1155	< .001
HS or less	22.7%	13.4%	10.7%	13.1%	
Some college	35.9%	27.4%	37.3%	35.2%	
College degree	27.6%	33.2%	31.2%	31.0%	
Post college	13.8%	26.0%	20.9%	20.8%	
Maternal race/ethnicity, n =	182	221	749	1152	< .001
Hispanic	25.3%	10.9%	12.4%	14.1%	
Black, non-Hispanic	12.6%	10.0%	6.8%	8.3%	
Other, non-Hispanic^a^	9.9%	11.8%	7.2%	8.5%	
White, non-Hispanic	52.2%	67.4%	73.6%	69.0%	
Parity, n =	182	223	752	1157	0.013
One	40.1%	48.9%	48.4%	47.2%	
Two	35.2%	37.7%	29.7%	32.1%	
Three	12.6%	8.1%	13.7%	12.4%	
Four or more	12.1%	5.4%	8.2%	8.3%	
Infant/toddler sex, n =	203	224	731	1158	0.496
Male	49.2%	52.7%	54.1%	53.0%	
Female	50.8%	47.3%	45.9%	47.0%	
Infant/toddler age, n =	203	224	731	1158	0.288
0–4 months	9.9%	11.6%	11.5%	12.0%	
5–8 months	15.3%	20.5%	14.1%	15.5%	
9–12 months	21.7%	20.1%	18.9%	19.6%	
13–24 months	53.2%	47.8%	55.5%	53.6%	

^**+**^P values reflect χ^2^ tests comparing use groups for each demographic characteristic

^a^Other, non-Hispanic categories: (1) Asian, (2) American Indian/Native American/Alaskan Native, (3) Native Hawaiian or Other Pacific Islander, (4) Other

### Crib Injury Risks

**Fig. 2:**
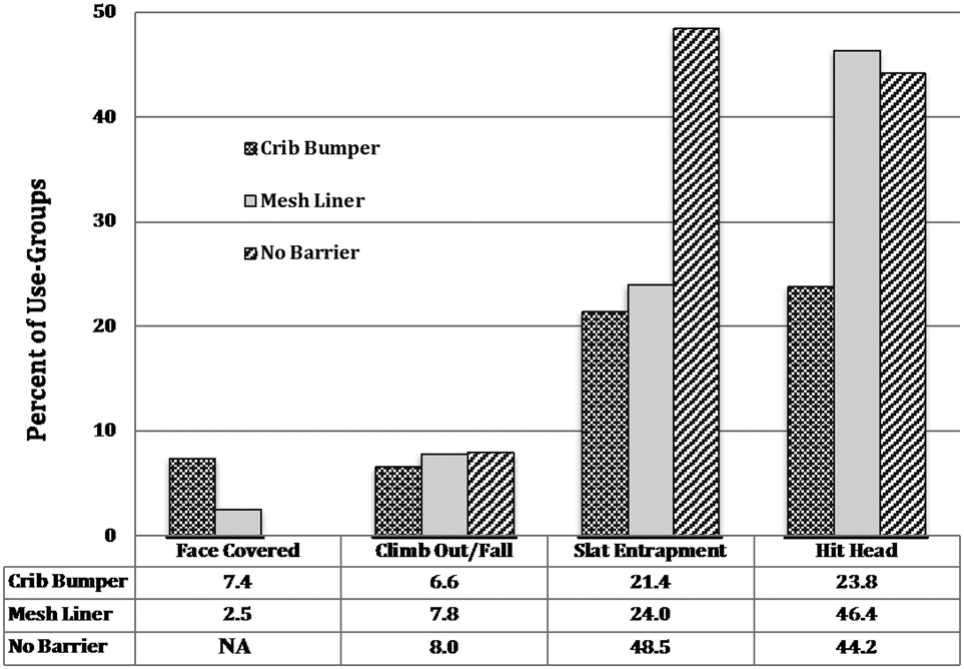
Percent of mothers reporting crib injury risks by use-group

For each crib injury risk, we calculated uORs and aORs adjusting for demographic variables with MCMC imputation for missing data (Table 2; Fig. 2). There was little difference between the unadjusted and MCMC-adjusted odds ratios for the four injury risks.

**Table 2:** Adjusted (aOR) and unadjusted (uOR) odds ratios for four crib injury risks

Characteristics^**+**^ (reference group)	Face-covered		Climb-out/fall		Slat-entrapment		Hit-head	
	aOR (95% CI)	p	aOR (95% CI)	p	aOR (95% CI)	p	aOR (95% CI)	p
Infant/toddler age^a,b^	0.47 (0.31–0.71)	< 0.01	2.20 (1.60–3.02)	< 0.01	0.76 (0.68–0.85)	< 0.01	0.69 (0.62–0.78)	< 0.01
Infant/toddler sex (female)	1.34 (0.52–3.47)	0.54	1.31 (0.82–2.09)	0.26	0.90 (0.71–1.14)	0.38	0.86 (0.67–1.09)	0.21
Mother age^a^	1.10 (0.67–1.80)	0.71	0.73 (0.56–0.95)	0.02	1.04 (0.90–1.20)	0.58	1.15 (0.99–1.33)	0.06
Mother race/ethnicity (White non-Hispanic)								
Hispanic	1.81 (0.54–6.02)	0.33	1.45 (0.78–2.72)	0.24	0.88 (0.62–1.26)	0.49	1.04 (0.72–1.48)	0.85
Black non-Hispanic	0.35 (0.04–2.90)	0.33	1.41 (0.63–3.13)	0.40	0.40 (0.24–0.67)	< 0.01	0.38 (0.23–0.63)	< 0.01
Other non-Hispanic	1.46 (0.37–5.72)	0.58	1.31 (0.60–2.86)	0.49	1.11 (0.72–1.71)	0.64	1.64 (1.04–2.57)	0.03
Mother education^a^	0.91 (0.55–1.51)	0.71	1.32 (0.99–1.74)	0.06	0.99 (0.85–1.14)	0.85	0.98 (0.84–1.13)	0.73
Parity^a^	1.23 (0.74–2.03)	0.42	1.50 (1.17–1.91)	< 0.01	1.00 (0.87–1.15)	0.99	0.90 (0.78–1.04)	0.15
Crib bumper (mesh liner)	3.52 (1.26–9.81)	0.02	NA	–	NA	–	NA	–
Crib bumper (no barrier)	NA	–	0.71 (0.38–1.35)	0.30	0.28 (0.19–0.40)	< 0.01	0.38 (0.26–0.54)	< 0.01
Mesh liner (no barrier)	NA	–	1.10 (0.64–1.92)	0.73	0.32 (0.23–0.44)	< 0.01	0.96 (0.71–1.31)	0.82

	uOR (95% CI)	p	uOR(95% CI)	p	uOR (95% CI)	p	uOR (95% CI)	p
Crib bumper (mesh liner)	3.06 (1.15–7.35)	0.03	NA	–	NA	–	NA	–
Crib bumper (no barrier)	NA	–	0.81 (0.44–1.47)	0.58	0.29 (0.20–0.41)	< 0.01	0.39 (0.28–0.56)	< 0.01
Mesh liner (no barrier)	NA	–	0.97 (0.57–1.65)	0.99	0.34 (0.24–0.46)	< 0.01	1.09 (0.82–1.46)	0.60

^a^Infant/toddler age, mother age, mother education, parity entered as ordinal variables

^b^Infant/toddler age at incident for each risk; otherwise age at time of survey

A total of 21 mothers (4.8%) reported their infant/toddler had been found at least once with his/her face covered. Of these, 15 infants/toddlers (7.4%) had their faces covered by CBs and 6 infants/toddlers (2.5%) by MLs. Mothers using CBs had 3.5 times higher adjusted odds (aOR) of finding their infant/toddler with face-covered than mothers using MLs (uOR = 3.1). Infants/toddlers with CBs were found with faces pressed against a crib bumper (n = 9), over or under crib bumpers, and wedged between a crib bumper and mattress or slats (n = 5). Two mothers reported their infants/toddlers’ faces were pressed against the bumper, had difficulty breathing and that the infants/toddlers’ faces were red or blue. One infant/toddler was found with face under the bumper and wedged between the slats and mattress. All three infants/toddlers were taken to a doctor/emergency room (ER). Six mothers reported their infants/toddlers’ faces pressed against MLs. No mother using MLs reported breathing difficulties, that their infant/toddler’s face turned red, blue, or was wedged. None took their infant/toddler to a doctor/ER.

Climb-out/falls, reported by 98 mothers (7%), showed no significant difference between CB versus NB and ML versus NB. No infant aged 0–4 months was reported to climb-out/fall. Four infants with CBs, 2 with MLs, and 1 with NB climbed-out/fell at 5–8 months old. Mothers reported injuries from climb-outs/falls, most from hit-heads or bruises (n = 28); a head injury and overnight hospital stay; a facture, and ten mothers took their infant/toddler to a doctor/ER.

Slat entrapment was reported by 494 mothers (37%). Mothers using CBs and MLs were significantly less likely to report slat-entrapments than mothers using NB (CB vs. NB aOR = .28, uOR = .32; and ML vs. NB aOR = .29, uOR = .34). Reports of injuries were mostly red marks; ten infants/toddlers went to a doctor/ER; a few reported minor injuries; and one fracture.

Hitting-heads on the crib sides was reported by 510 mothers (38%). Mothers using CBs were significantly less likely to report hit-heads compared to mothers using NB (aOR = .38, uOR = .39). There was no significant difference in reports of hit-heads for mothers using MLs and NB (aOR = .96, uOR = 1.09). Mothers reported bruises and 12 mothers reported cuts and scrapes. No infant/toddler was taken to a doctor/ER.

Several demographic characteristics were significantly associated with injury risks. Older infants/toddlers were more likely to have reports of climb-out/falls. Younger infants/toddlers were more likely to have reports of face-covered, slat-entrapment, and hit-heads. White non-Hispanic mothers were more likely to report slat entrapments and hit-heads compared to black non-Hispanic mothers; and other non-Hispanic mothers were more likely to report hit-heads compared to white non-Hispanic mothers. Younger mothers were more likely to report climb-out/falls.

### Reasons for Barrier/No Barrier Choice

Most mothers using CBs and MLs gave high ratings to preventing slat-entrapments (88.8% and 91.4%) as a reason for their choice while significantly more mothers using CBs than MLs gave high ratings to preventing hit-heads (93.5% vs. 46.1%). Mothers who chose NB gave high ratings to “I don’t worry about…arms/legs in the crib slats (43.3%) and “I don’t worry about…hitting his/her head” (49.6%) as reasons for their choice, suggesting that many NB mothers did worry about these minor risks but continued to use no-barrier (Table 3).

**Table 3:** Reasons for choice of barrier/no barrier use: strongly agree/agree

CB/ML: I use a [CB, ML] in my baby’s crib because NB: I chose to use nothing in my baby’s crib because	Crib bumper % (N^a^)	Mesh liner % (N^a^)	p^+^	No barrier % (N^a^)
Suffocation: There is no suffocation risk with a (CB, ML).	40.6% (86)	64.1% (157)	< .001	
Suffocation: (NB) I was concerned about suffocation risk.				83.6% (681)
Safe: (CB, ML) They are safe.	59.1% (124)	81.2% (199)	< .001	
Safe: (NB) It’s safer to use nothing in the crib.				88.7% (723)
Slats: The (CB, ML) prevents my baby from getting his/her arms/legs caught in the crib slats.	88.8% (190)	91.4% (223)	.928	
Slats: (NB) I don’t worry about my baby getting his/her arms/legs in the crib slats.				43.3% (353)
Hit Head: The (CB, ML) prevents my baby from hitting his/her head against the crib.	93.5% (200)	46.1% (113)	< .001	
Hit Head: (NB) I don’t worry about my baby hitting his/her head.				49.6% (404)
Other: I already had a (CB, ML).	72.8% (155)	44.2% (108)	< .001	
Other: (CB, ML) are cute and decorate my baby’s room.	62.5% (133)	30.4% (74)	< .001	

^**+**^p values reflect χ^2^ tests comparing CB, ML for each rating

^a^Number reporting this response

Significantly more mothers using MLs than CBs gave high ratings to safety as a reason for their choice (81.2% vs. 59.1%) and to “there is no suffocation risk” (64.1%, vs. 40.6%). Most mothers using NB gave high ratings to both safety and concerns about suffocation risk (88.7% and 83.6%). Finally, significantly more mothers using CBs than MLs gave high ratings to availability, “I already had one” (72.8% vs. 44.2%) and aesthetics “They’re cute and decorate my baby’s room” (62.5% vs. 30.4%) as reasons for their choice.

## Discussion

This is the first study to compare the relative safety of crib bumpers, mesh liners, and no barrier in the crib by examining four injury risks for a general population of mothers. To our knowledge, this is also the first study to examine mothers’ reasons for their choice of crib bumpers, mesh liners or nothing in the crib and the prevalence of those choices.

The most serious injury risk was face-covered because of the potential for suffocation. CB mothers reported breathing difficulties, wedging, and infants/toddlers with face pressed against the bumper, similar to circumstances that have also caused deaths (Scheers et al. 2016; Thach et al. 2007). However, 41% of mothers using crib bumpers rated “no suffocation risk” and 59% rated “they are safe” highly as reasons for their choice, indicating a lack of awareness that crib bumpers are potentially dangerous.

Relative to no barrier use, mothers using crib bumpers reported fewer slat-entrapments and hit-heads, both reasons most mothers using crib bumpers chose to use them (Joyner et al. 2009). However, it is fair to assume that the risk of suffocation from crib bumper use outweighs any benefit from preventing minor injuries (Yeh et al. 2011) and it is unlikely that an infant/toddler could exert enough force in hitting its head to produce anything other than a minor injury (Thach et al. 2007).

While mothers using mesh liners also found their infant/toddler with their faces pressed against the liner, there was no breathing difficulty or wedging reported, likely because liners are thin and do not trap exhaled air. While there were fewer slat-entrapments with mesh liners similar to crib bumpers, there was no significant difference in reports of infants/toddlers hitting their heads on the crib sides between mesh liners and no barriers. Most mothers using mesh liners rated preventing slat entrapment (91%) and no suffocation risk (64%) highly as reasons for their choice.

Most mothers in our study used no barrier in the crib (63%), suggesting that the AAP’s recommendation to have nothing in the crib is effective, at least for this population of mothers. Despite the fact that infants/toddlers with no barrier experienced higher rates of slat-entrapment and hit-heads on the crib sides, most of these mothers rated safety and no suffocation risk highly as reasons for their choice. However, one mother using no barrier commented that slat-entrapment caused her to put a bumper in the crib.

The rates of climb-outs/falls showed no significant difference among the use-groups adjusted for infant/toddler age and other demographics. Similar to findings by Yeh et al. (2011), our study found that older infants/toddlers were more likely to climb-out/fall. In both studies, details were lacking as to how they climbed out other than by simple dexterity (“She just flipped out”). Other examples include: “I had a thick blanket and crib wasn’t lowered she crawled over” (mother using no barrier). “She crawled out using the crib and mattress for leverage” (mother using a mesh liner). “I witnessed her step on top of the bumpers many times to elevate herself” (mother using a crib bumper).

Reducing crib bumper use is challenging. There is widespread use of bumper images in the media and displays in stores (Joyner et al. 2009; Kreth et al. 2017) implying that bumpers are safe. Along with other studies (Colvin et al. 2014; Caraballo et al. 2016), we found that many mothers are unaware of the dangers of crib bumpers, with 59% of crib bumper mothers in our study choosing crib bumpers because “they are safe.^”^ Advising parents to avoid soft bedding, including crib bumpers, to prevent SIDS may be a confusing and mixed message for many parents. For example, the recent “Safe to Sleep Campaign” (n.d.) messages include “…SIDS …doesn’t have a known cause even after a complete investigation” and “SIDS is not the same as suffocation and is not caused by suffocation” but note that soft bedding is a risk factor for SIDS. Pease et al. (2017) in the United Kingdom found that mothers followed the more straightforward advice to remove soft bedding to avoid suffocation.

Our study provides insights into the complex task of counseling parents and devising targeted interventions in a relatively well-educated population compared to the U.S. population (Martin et al. 2017). We found that most mothers who use crib bumpers believe they are providing a safe sleep environment by preventing slat-entrapments and hitting-heads on the crib sides. These crib risks, resulting primarily in minor injuries, are much more common experiences among parents while potentially deadly events from face-covered and wedging are much less common. Minimizing the potential for hitting-heads requires sufficient padding that is also a potential suffocation hazard, but slat-entrapment may be minimized through alternative products. Another option for parents is to use a sleep sack with nothing else in the crib, although 16.5% of mothers with no barrier in our survey who “usually” or “sometimes” used sleep sacks also reported slat-entrapment.

### Limitations

First, our results may not generalize to a general population of mothers because mothers in our survey who subscribed to a parenting magazine were more likely to: have ≥ college degree (52% vs. 29%) (Livingston and Cohn 2013), be White non-Hispanic (69% vs. 53.5%); and ≥ 35 years old (26% vs. 16%) (Martin et al.  2017). Also, limiting the respondents to mothers as caregivers may miss a diversity of practices among father/grandfather/other caregivers. Second, our results may have been affected by the response rate and be biased even for the population of parenting magazine subscribers. However, our finding of 17% crib bumper use is close to the overall estimate of 19% for 13 states and NYC (Bombard et al. 2018). Third, mothers’ self-report of their infant/toddler’s injuries may be biased in reporting those events. Studies found that highly negative events may be remembered more accurately, and to the extent that crib injuries are highly negative events, there may be less bias than in other recall situations (Bowen et al. 2018). Fourth, we do not have details on what other objects (blankets, pillows, etc.) may have been present in the sleeping environment or the height of the crib mattress, especially important when interpreting results for climb-outs/falls. Finally, imputation for missing data may have affected results although unadjusted and MCMC-adjusted odds ratios are very similar.

## Conclusions for Practice

We surveyed mothers who subscribed to a national parenting magazine that focuses on issues of interest to individuals with infants and children. Despite this, there was frequent use of crib bumpers, a product known to be dangerous to infants/toddlers, with breathing difficulties and wedgings reported. Mothers using no barriers or mesh liners did not report these problems. Many mothers were more concerned about preventing minor risks of slat-entrapment and hit-heads than suffocation. Understanding reasons for mothers’ use of barriers/no barriers is important for pediatricians and other public health practitioners in tailoring counseling for mothers with infants/toddlers and educating them about safe sleep practices.

## Electronic supplementary material

Below is the link to the electronic supplementary material.

Supplementary material 1 (DOCX 14 kb)
