# Supplementary material for: Reports of Injury Risks and Reasons for Choice of Sleep Environments for Infants and Toddlers
Source: Matern Child Health J. 2019 Jun 27;23(12):1613–20. doi: 10.1007/s10995-019-02803-7 (PMC6823298; doi:10.1007/s10995-019-02803-7)
Supplement: Supplementary file 1 — Supplementary material 1 (DOCX 14 kb) [file 10995_2019_2803_MOESM1_ESM.docx]

Appendix 1: Survey Questions for Crib Injuries

| *Questions for Climb-Out/Fall, Slat Entrapment, Hit Head*  A. Did your baby ever climb out and fall from a crib?  A. Did you ever find your baby got their legs or arms caught in the   crib slats?  A. Did your baby ever hit his/her head on the crib sides?  *Instructions: If your baby [injury incident] more than once, please answer the next few questions for the time that worried you the most.*  B. Did the [incident] cause your baby to get: [appropriate list for   each injury type]: Hit head, head injury, bruise, red marks,   swollen arm or leg, cut or scrape, fracture, another injury?  C. Did you take your baby to the doctor or emergency room?  D. If your baby was taken to the emergency room, was he/she kept in  the hospital overnight?  E. About how old was your baby when [injury incident] occurred? | *Response Options*  A. Yes once;  Yes, more than once;  No  B. Yes;   No for each injury  C. Yes; No  D. Yes; No; Not taken   to emergency room  E. Drop down menu in   months |
| --- | --- |
| *Questions for Face-Covered*  A. Did you ever find your baby with his/her face partially or fully   covered by a [crib bumper/mesh liner]?  *Instructions: If your baby got his/her face covered by a [CB/ML] more than once, please answer the next few questions for the time that worried you the most.*  B. How was your baby’s face covered?  Face was pressed against the [CB, ML].  [CB, ML] was over the baby’s face.  Baby was under the [CB, ML].  Baby was stuck between the [CB, ML] and the crib slats.  Baby was stuck between the [CB, ML] and the mattress.  Baby was stuck between the [CB, ML] and another object.  Other (please specify).  C. When the [CB, ML] was covering your baby’s face, did you see   any signs that your baby had difficulty breathing?  D. Was any part of your baby’s face red or blue from not being able  to breathe?  E. Did you take your baby to a doctor or emergency room?  F. About how old was your baby when his/her face became covered   by the [CB/ML]? | *Response Options*  A. Yes; No  B. Yes; No  C. Yes; No; Not Sure  D. Yes; No; Not Sure  E. Yes; No  F. Drop down menu in   months |
